# Effect of salt intake on beat‐to‐beat blood pressure nonlinear dynamics and entropy in salt‐sensitive versus salt‐protected rats

**DOI:** 10.14814/phy2.12823

**Published:** 2016-06-10

**Authors:** Souha A. Fares, Joseph R. Habib, Milo C. Engoren, Kamal F. Badr, Robert H. Habib

**Affiliations:** ^1^ Hariri School of NursingAmerican University of BeirutBeirutLebanon; ^2^ Vascular Medicine Program and Department of Internal MedicineAmerican University of BeirutBeirutLebanon; ^3^ Department of AnesthesiologyUniversity of MichiganAnn ArborMichigan; ^4^ Outcomes Research Unit – Clinical Research InstituteAmerican University of BeirutBeirutLebanon

**Keywords:** Beat‐to‐beat variability, complexity, detrended fluctuation analysis

## Abstract

Blood pressure exhibits substantial short‐ and long‐term variability (BPV). We assessed the hypothesis that the complexity of beat‐to‐beat BPV will be differentially altered in salt‐sensitive hypertensive Dahl rats (SS) versus rats protected from salt‐induced hypertension (SSBN13) maintained on high‐salt versus low‐salt diet. Beat‐to‐beat systolic and diastolic BP series from nine SS and six SSBN13 rats (http://www.physionet.org) were analyzed following 9 weeks on low salt and repeated after 2 weeks on high salt. BP complexity was quantified by detrended fluctuation analysis (DFA), short‐ and long‐range scaling exponents (*α*_S_ and *α*_L_), sample entropy (SampEn), and traditional standard deviation (SD) and coefficient of variation (CV(%)). Mean systolic and diastolic BP increased on high‐salt diet (*P* < 0.01) particularly for SS rats. SD and CV(%) were similar across groups irrespective of diet. Salt‐sensitive and ‐protected rats exhibited similar complexity indices on low‐salt diet. On high salt, (1) SS rats showed increased scaling exponents or smoother, systolic (*P* = 0.007 [*α*_L_]) and diastolic (*P* = 0.008 [*α*_L_]) BP series; (2) salt‐protected rats showed lower SampEn (less complex) systolic and diastolic BP (*P* = 0.046); and (3) compared to protected SSBN13 rats, SS showed higher *α*_L_ for systolic (*P* = 0.01) and diastolic (*P* = 0.005) BP. Hypertensive SS rats are more susceptible to high salt with a greater rise in mean BP and reduced complexity. Comparable mean pressures in sensitive and protective rats when on low‐salt diet coupled with similar BPV dynamics suggest a protective role of low‐salt intake in hypertensive rats. This effect likely reflects better coupling of biologic oscillators.

## Introduction

Blood pressure (BP) exhibits substantial natural variability that ranges from beat‐to‐beat differences, within‐the‐day differences, and possibly across days or longer periods (Parati et al. 2013). BP variability (BPV) may be modified with processes such as aging, external stress, diet, as well as cardiovascular disease or hypertension. Multiple studies have shown that indices of BPV may independently predict organ damage and cardiovascular morbid and fatal events in certain patient groups (Parati et al. 2013). Relatedly, changes in BPV properties potentially have important consequences on treatment, yet these remain poorly understood.

Traditionally, the standard deviation (SD) and the coefficient of variation (CV) of the systolic, diastolic, or mean BP time series have been used to assess BPV. However, other more sophisticated techniques such as complexity analysis (Pincus 1991) and detrended fluctuation analysis (DFA) (Peng et al. 1995) have been introduced for the analysis of beat‐to‐beat variability of physiologic data. These nonlinear dynamical measures were shown to be effective in predicting organ damage and cardiovascular events in a manner complementary to the traditional measures and the mean BP (Ho et al. 1997; Au‐Yeung et al. 2015; Moridani et al. 2015). They also showed trends in the variability of biological signals that traditional variability measures failed to detect (Subramaniam et al. 2014; Vandendriessche et al. 2014).

Indices of signal complexity such as entropy measures (e.g., sample entropy [SampEn], approximate entropy [ApEn]) quantify the irregularity of biological signals; the more regular the signal, the lower the entropy value (Pincus 1991; Richman and Moorman 2000). Alternatively, DFA quantifies the correlation properties of the beat‐to‐beat time series via one or more scaling exponents (*α*) using the fractal property; more regular systems are characterized by higher values of *α* (Goldberger et al. 2002; Golinska 2012). Healthy organisms are characterized by higher degrees of complexity (Seely and Christou 2000) and therefore by higher entropies and lower scaling exponents. Complexity and DFA methods have been effectively used in analysis of heart and, to a lesser extent, respiratory rate variability (Valencia et al. 2009; Ernst and Rostrup 2010; Frey et al. 2011; Ho et al. 2011; Tejera et al. 2012; Cancio et al. 2013; Mejaddam et al. 2013; Weippert et al. 2013). Their use, however, in the analysis of blood pressure variability remains very limited.

This study aimed to explore the potential use of entropy (complexity) and fractal scaling exponents (correlation properties via DFA) for informative analysis of beat‐to‐beat blood pressure (BP) data from salt‐sensitive hypertensive Dahl rats (SS) and rats protected from salt‐induced hypertension (SSBN13). Toward this, we derived and compared the short‐ and long‐term correlation properties (DFA scaling coefficients: *α*_S_ and *α*_L_) and SampEn parameters under different conditions of hemodynamic stress modeled via exposure to high‐salt versus low‐salt diet. An implied hypothesis was that salt‐sensitive hypertensive rats will exhibit different nonlinear BP dynamics compared to protected rats, irrespective of diet, as well as show a differential impact of salt intake on these metrics across the two strains of rats.

## Materials and Methods

### Animals and experimental protocol

We derived beat‐to‐beat BP series data from nine salt‐sensitive hypertensive (SS) Dahl rats and six rats protected from high‐salt‐induced hypertension (SSBN13) for whom raw sampled BP data epochs are available on Physionet (Goldberger et al. 2000). These data were previously analyzed to investigate the physiological origins of the baroreflex dysfunction in the SS rats (Bugenhagen et al. 2010). The original study was approved by the Institutional Animal Care and Use Committee of the Medical College of Wisconsin (Bugenhagen et al. 2010). Briefly, the SSBN13 rats were derived by interbreeding two strains: the hypertensive SS rats and the normotensive Brown Norway rats (BN) as described previously (Cowley et al. 2001). Rats were maintained on a low‐salt (0.4% salt) diet for 9 weeks. After a 1‐week recovery period, the diet was switched to high salt (8% salt) for 2 weeks. Blood pressure was collected at each salt level using radiotelemetry for 2 min sampled at 100 Hz. Two‐minute data epochs were available (Goldberger et al. 2000 ) for all rats at each salt level. We derived beat‐to‐beat systolic and diastolic BP series for analysis using peak (valley) detection methods. The 2‐min time series of the BP parameters varied between 600 and 900 beats in length (*N*).

### Detrended fluctuation analysis

Detrended fluctuation analysis (DFA) has been developed to detect the correlations of varying ranges (short and long range) embedded in a time series using the fractal property (Peng et al. 1995). First, the original time series of total length *N* is integrated, yielding:

(1) $$y\left(k\right)=\sum_{i=1}^{k}\left(y\left(i\right)-y_{m}\right)$$

where *y*(*i*) is the *i*th beat‐to‐beat value and *y*_m_ is the mean value. The integrated time series is then divided into boxes of equal length *n* and *y*(*k*) is detrended by removing the local trend, *y*_*n*_(*k*), consisting of the least square linear fit of each box. The root mean square fluctuation of the detrended time series *F*(*n*) is calculated over different box sizes (*n*), and hence providing a relationship between *F*(*n*) and *n*:

(2) $$F\left(n\right)=\sqrt{\frac{1}{N}\sum_{k=1}^{N}{\left(y\left(k\right)-y_{n}\left(k\right)\right)}^{2}}$$

The linear slope of the log_10_ *F*(*n*) to log_10_ *n* is the DFA scaling exponent *α*. As used previously (Peng et al. 2002; Castiglioni et al. 2011; Zhang et al. 2013), DFA can be analyzed in two ways: a single slope *α* for the whole time series and double slopes, one for short‐term correlations (*α*_S_) and one for long‐term correlation (*α*_L_) based on an a priori cutoff (*n *=* *10).

For our SBP and DBP time series we used the double slope approach as it better reflected our data. To have a minimum of six windows averaged, we analyzed DFA up to a window size of *n *=* *100. The short‐range slope *α*_S_ was computed for *n *=* *4–10 and the long‐range slope *α*_L_ for *n *=* *11–100. DFA scaling exponents *α*_S_ and *α*_L_ are measures of the smoothness of a time series, higher correlations indicate increased predictability or smoother series (Peng et al. 1995). *α*_S_ and *α*_L_ of SBP and DBP were computed for datasets of the two strains of rats on high‐ and low‐salt diets using a Matlab (MathWorks, Natick, MA) code implemented in our laboratory.

### Sample entropy analysis

Sample entropy (SampEn) is a measure of the self‐similarity in a time series (Richman and Moorman 2000). It depends on two parameters: a dimension *m* which is the number of consecutive data points in a pattern and a tolerance *r* within which the *m* points are considered a self‐match. SampEn is the negative natural logarithm of the conditional probability that sequences within *r* for the *m* consecutive data points and remain within *r* for the next point. SampEn is related to ApEn (Pincus 1991), but unlike it, SampEn eliminates self‐counting of matches and as a result minimizes the dependency of this complexity index on the length of the time series (Richman and Moorman 2000). Using SampEn will thus remove this bias introduced by ApEn. SampEn was calculated for *m *=* *2 and *r *=* *0.2. Low values of SampEn indicate loss of complexity and more predictability (more consistent with abnormal BP dynamics). SampEn of SBP and DBP was computed for all rats on both diets using a Matlab code available on Physionet (http://www.physionet.org/physiotools/sampen/c/).

### Statistical analyses

In addition to scaling exponents (*α*_S_, *α*_L_) and SampEn, we derived the standard deviation (SD) and coefficient of variation (CV(%)) for SBP and DBP time series. Continuous variables are presented as means ± standard error (SE) and were compared across groups and salt diets using the independent samples *t* test (between group) or the paired *t* test (within group) in case of normally distributed data. Alternatively, in case of non‐normal data, the nonparametric Mann–Whitney *U* test or the signed‐rank test were used. *P*s < 0.05 were considered significant. Statistical analyses were performed using SigmaPlot version 11.2 for Windows.

## Results

**Figure 1:**
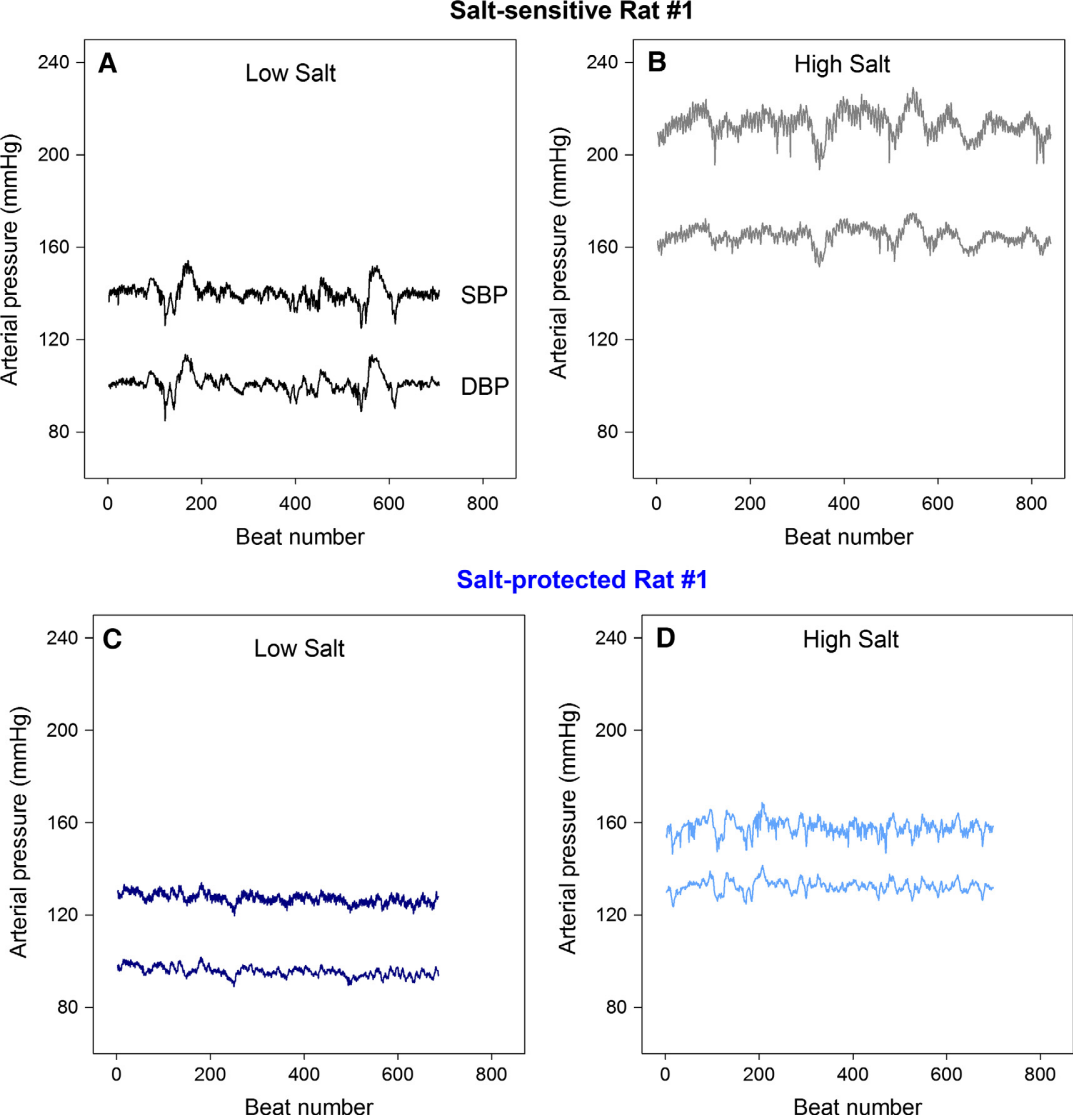
Example of beat‐to‐beat systolic (SBP) and diastolic (DBP) blood pressure time series derived from 2‐min data epoch recordings: (A) salt‐sensitive rat on low‐salt diet; (B) same salt‐sensitive rat on high‐salt diet; (C) protected rat on low‐salt diet; and (D) same protected rat on high‐salt diet.

Beat‐to‐beat systolic (SBP) and diastolic (DBP) time series, for example, rats belonging to the two groups on both diets, are shown in Figure 1. On low‐salt diet, salt‐sensitive hypertensive rats exhibited higher mean systolic pressure compared to the protected rats (137 ± 2 vs. 125 ± 2, *P* = 0.006), while the mean diastolic pressures were similar (Table 1). As expected, mean systolic and diastolic blood pressure increased significantly with high salt, but more so in the salt‐sensitive hypertensive rats – SBP (salt sensitive vs. protected): Δ_H‐L_ = 57 ± 5 versus Δ_H‐L_ = 30 ± 2, *P* = 0.002; DBP (salt sensitive vs. protected): Δ_H‐L_ = 51 ± 6 versus Δ_H‐L_ = 26 ± 3, *P* = 0.003 (Table 1). The standard deviation and coefficient of variation were similar for systolic and diastolic blood pressure time series across groups and irrespective of diet (Table 2).

**Table 1:** Comparison of salt‐intake effects on BP in salt‐sensitive and ‐protected rats

Study group/Treatment	Salt sensitive			Salt protected		
	Low salt	High salt	Δ_HS‐LS_	Low salt	High salt	Δ_HS‐LS_
Systolic BP (mmHg)	137 ± 2^a^, ^b^	195 ± 7^a^, ^b^	57 ± 5^b^	125 ± 2^a^, ^b^	155 ± 3^a^, ^b^	30 ± 2^b^
Diastolic BP (mmHg)	102 ± 3^a^	153 ± 7^a^, ^b^	51 ± 6^b^	97 ± 1^a^	123 ± 3^a^, ^b^	26 ± 3^b^

Number of beats: 626–953; data shown as mean ± standard error averaged across rats within a group; ^a^ *P *< 0.05 low salt versus high salt (within group); ^b^ *P *< 0.05 salt sensitive versus salt protected (between groups; same treatment).

Δ_HS‐LS_: changes calculated as (high‐salt − low‐salt) values.

**Table 2:** Comparison of traditional BP variability metrics in salt‐sensitive and ‐protected rats

Study group/Treatment	Salt sensitive			Salt protected		
	Low salt	High salt	Δ_HS‐LS_	Low salt	High salt	Δ_HS‐LS_
Systolic BP						
SD (mmHg)	4.40 ± 0.30	5.73 ± 0.46	1.33 ± 0.57	4.30 ± 1.30	5.19 ± 0.49	0.89 ± 0.59
CV (%)	3.2 ± 0.2	2.9 ± 0.2	−0.30 ± 0.34	3.5 ± 0.4	3.4 ± 0.4	−0.10 ± 0.48
Diastolic BP						
SD (mmHg)	4.03 ± 0.31	4.70 ± 0.48	0.67 ± 0.59	3.80 ± 0.50	4.65 ± 0.56	0.85 ± 0.58
CV (%)	3.97 ± 0.25	3.13 ± 0.29	0.85 ± 0.41	3.89 ± 0.47	3.82 ± 0.47	−0.08 ± 0.48

Number of beats: 626–953; data shown as mean ± standard error averaged across rats within a group; Δ_HS‐LS_: changes calculated as (high‐salt − low‐salt) values.

### Detrended fluctuation analysis

**Figure 2:**
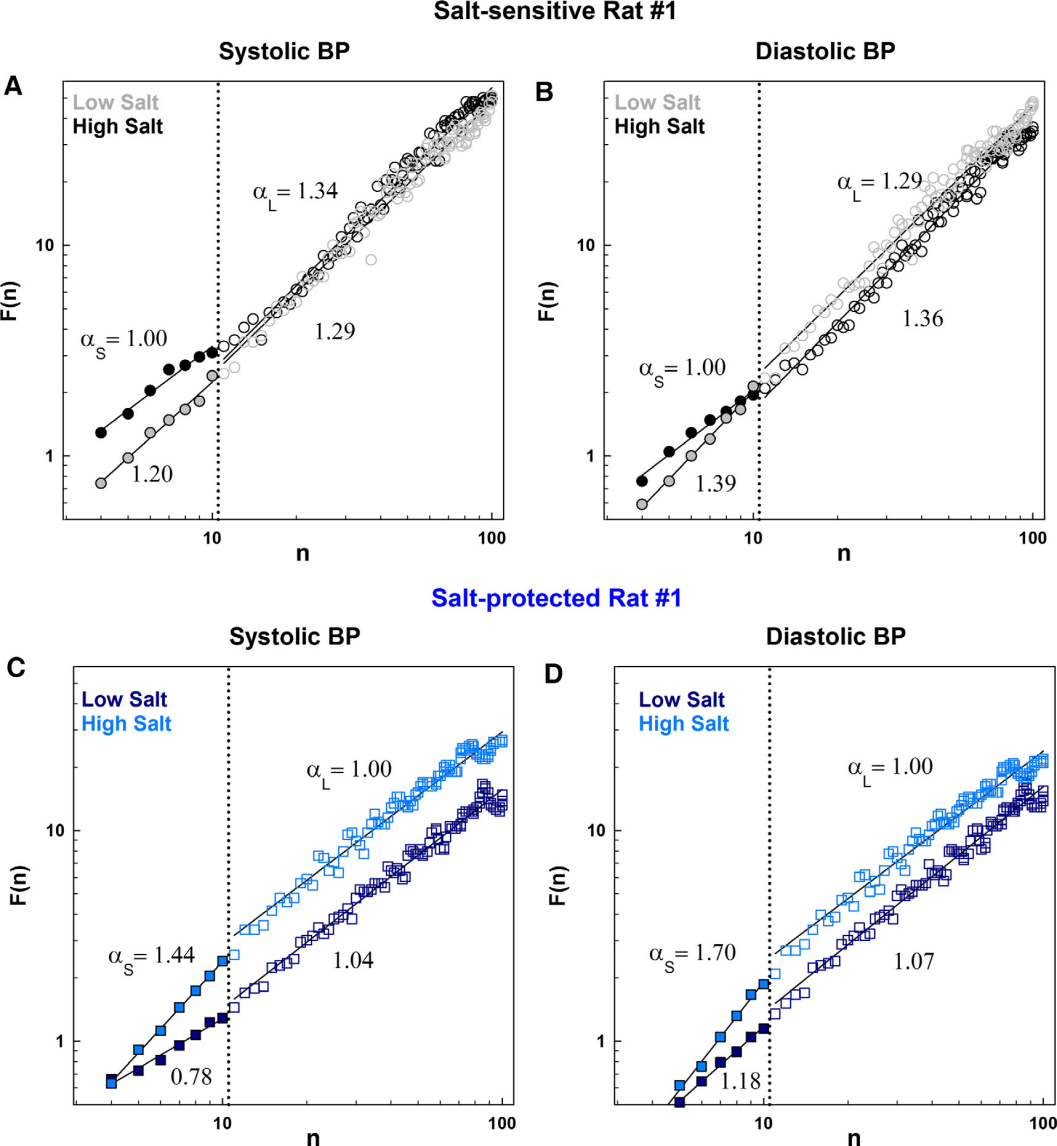
Log–log plot of DFA‐derived *F*(*n*) versus *n* derived for systolic and diastolic BP time series datasets shown in Figure 1, for example, salt‐sensitive and ‐protected rats while on low‐salt diet compared to high‐salt diet: (A) SBP, salt‐sensitive rat; (B) DBP, same salt‐sensitive rat; (C) SBP, protected rat; and (D) DBP, same protected rat. Log–log plots of all rats are shown in Figures S1–S4.

**Figure 3:**
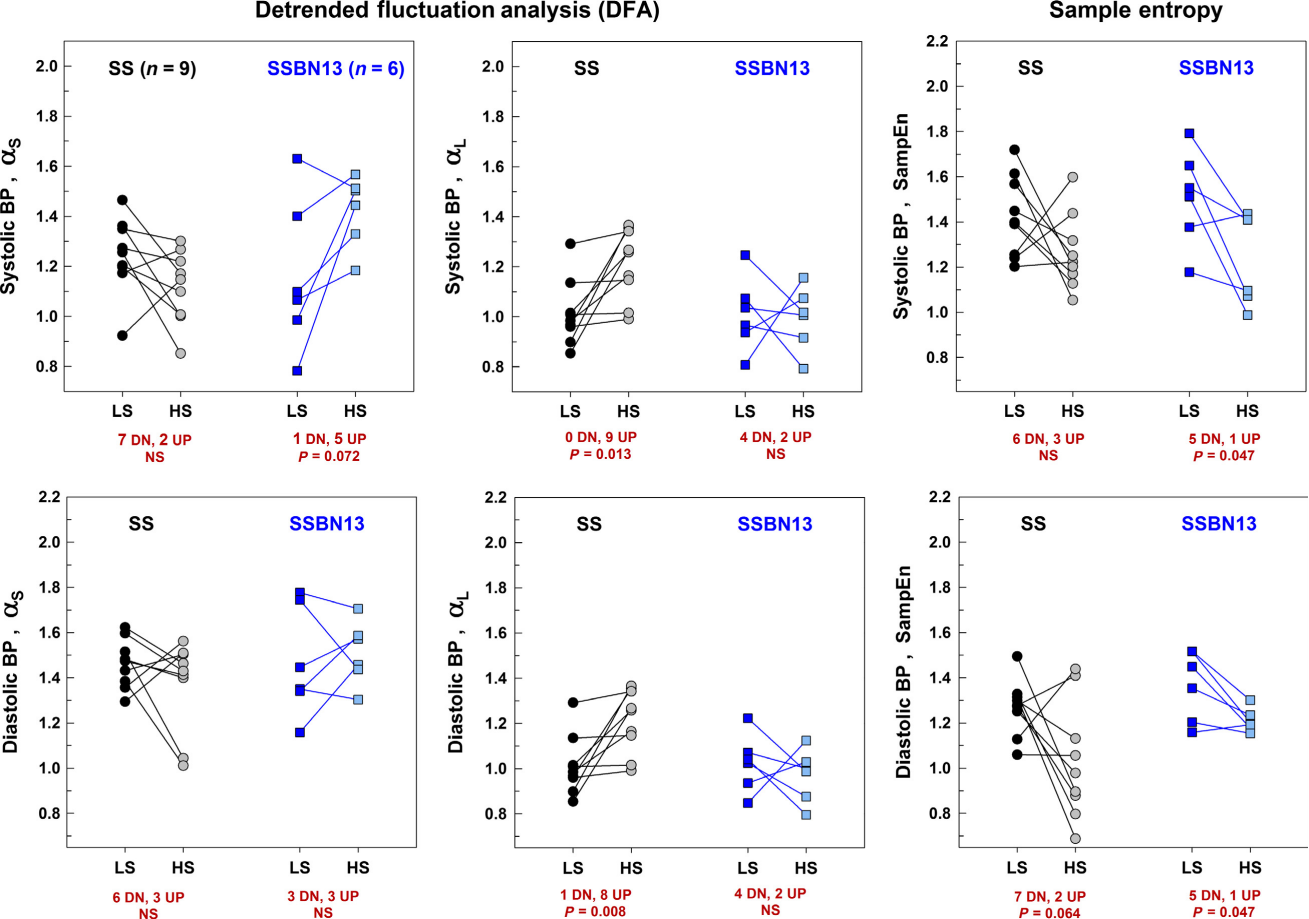
Effect of high‐salt (HS) diet following low‐salt (LS) diet on entropy and detrended fluctuation properties of the systolic and diastolic BP time series shown for all study salt‐sensitive (*n* = 9) and ‐protected (*n* = 6) rats individually: (A) *α*_S_ of SBP; (B) *α*_L_ of SBP; (C) SampEn of SBP; (D) *α*_S_ of DBP; (E) *α*_L_ of DBP; and (F) SampEn of DBP. Comparisons were done using the paired *t* test and the signed‐rank test as appropriate.

Examples of DFA log–log plot for SBP and DBP in the short‐term and long‐term beat ranges are shown in Figure 2. Table 3 summarizes the results of DFA scaling exponents *α*_S_ and *α*_L_. On low‐salt diet, both strains of rats exhibited similar systolic and diastolic scaling exponents. High‐salt diet altered the blood pressure scaling exponents in both the salt‐sensitive and salt‐protected rats (Table 3 and Fig. 3): salt‐sensitive rats showed increased *α*_L_ (smoother) for systolic BP (*P* = 0.012 and *P* = 0.007, respectively) and diastolic BP (*P* = 0.013 and *P* = 0.008, respectively); protected rats showed a trend toward an increased *α*_S_ for systolic BP (*P* = 0.072). On high‐salt diet, salt‐sensitive rats showed higher *α*_L_ for systolic and diastolic BP (*P* = 0.01 and *P* = 0.005, respectively) and lower *α*_S_ for systolic BP (*P* = 0.001) compared to the protected rats.

**Table 3:** Comparison of entropy and detrended fluctuation analysis indices in salt‐sensitive and ‐protected rats

Study group/Treatment	Salt sensitive			Salt protected		
	Low salt	High salt	Δ_HS‐LS_	Low salt	High salt	Δ_HS‐LS_
Systolic BP						
Scaling exponents						
*α*_S_	1.24 ± 0.05	1.12 ± 0.05^b^	−0.13 ± 0.07^b^	1.16 ± 0.12aᵻ	1.43 ± 0.06aᵻ, b	0.27 ± 0.06^b^
*α*_L_	1.01 ± 0.04^a^	1.21 ± 0.05^a^, ^b^	0.20 ± 0.06bᵻ	1.01 ± 0.06	0.99 ± 0.05^b^	−0.02 ± 0.09bᵻ
Entropy						
SampEn	1.43 ± 0.06	1.26 ± 0.06	−0.16 ± 0.10	1.51 ± 0.09^a^	1.24 ± 0.08^a^	−0.28 ± 0.09
Diastolic BP						
Scaling exponents						
*α*_S_	1.46 ± 0.04	1.37 ± 0.07	−0.09 ± 0.08	1.47 ± 0.10	1.51 ± 0.06	0.04 ± 0.10
*α*_L_	1.04 ± 0.04^a^	1.21 ± 0.05^a^, ^b^	0.17 ± 0.05^b^	1.02 ± 0.05	0.97 ± 0.05^b^	−0.05 ± 0.08^b^
Entropy						
SampEn	1.27 ± 0.04aᵻ	1.03 ± 0.09aᵻ bᵻ	−0.24 ± 0.14	1.37 ± 0.06^a^	1.21 ± 0.02^a^, bᵻ	−0.16 ± 0.06

Number of beats: 626–953; data shown as mean ± standard error averaged across rats within a group; ^a^ *P *< 0.05 low salt versus high salt (within group); ^b^ *P *< 0.05 salt sensitive versus salt protected (between groups; same treatment); ^aᵻ^0.05 ≤ *P *<* *0.1 low salt versus high salt (within group); ^bᵻ^0.05 ≤ *P *<* *0.1 salt sensitive versus salt protected (between groups; same treatment). *α*_S_ for *N*_beats_ = 4–10; *α*_L_ for *N*_beats_ = 11–100.

Δ_HS‐LS_: changes calculated as (high‐salt − low‐salt) values.

### Sample entropy

Table 3 summarizes the results of SampEn. On low‐salt diet, both strains of rats exhibited similar systolic and diastolic complexity. High‐salt diet altered the blood pressure complexity in both strains (Table 3 and Fig. 3): salt‐sensitive rats showed a trend toward a decreased SampEn (less complex) for diastolic BP (*P* = 0.064); protected rats showed a decreased SampEn for systolic and diastolic BP (*P* = 0.047 and *P* = 0.037, respectively).On high‐salt diet, salt‐sensitive rats showed a trend toward a lower SampEn for diastolic BP (*P* = 0.087) compared to the protected rats (Table 3).

## Discussion

We found that on low‐salt diet, both groups of rats had similarly irregular or complex BP time series. However, when exposed to a high‐salt diet, both groups showed a loss of complexity with the salt‐sensitive rats showing a greater loss of complexity (Table 3). Whereas, standard measures of variability, that is, standard deviation and coefficient of variation (Table 2), did not differ. Our results are consistent with the study by Vandendriessche et al. where two mouse models were compared in terms of SD of the beat‐to‐beat diastolic blood pressure series after induced shock (Vandendriessche et al. 2014), and another study by Subramaniam et al. where the SD of the systolic, diastolic, and pulse pressure series of 20 subjects with preoperative major adverse events during surgery were compared to those of 20 matched controls (Subramaniam et al. 2014). The SD's were unchanged between cases and controls in both studies. Older studies that showed the effect of the SD and CV on prognosis and cardiovascular outcomes, such as stroke, myocardial infarction, and death, have analyzed BPV using ambulatory blood pressure monitoring over 24 h at fixed intervals such as every 5–15 min, 1 h, or between visits (Hansen et al. 2010; Rothwell et al. 2010a,b; Stolarz‐Skrzypek et al. 2010; Muntner et al. 2011). Recently, however, beat‐to‐beat BPV became of greater interest due to the advancements in noninvasive monitoring, which allows blood pressure data to be measured continuously (beat to beat) over epochs of 5–30 min or longer.

As first proposed about 30 years ago (Godin and Buchman 1996), physiological rhythms, such as BP, are under the control of coupled biological oscillators, which are affected by neural, humoral, and cytokine components. Changes in any of these components would then lead to changes in physiologic rhythms, which can be measured and quantitated. Salt loading increases both BP and microvascular permeability (Rorije et al. 2015). Salt loading acts on the V_1a_ receptors in the paraventricular nucleus of the hypothalamus to modulate the salt‐induced sympathoexcitation (Ribeiro et al. 2015). Furthermore, salt intake differentially effects muscle sympathetic nerve activity and catecholamine levels in salt‐sensitive and salt‐resistant persons (Miyajima and Yamada 1999). The renin–angiotensin–aldosterone system is partially effected by pulsatile secretion of these hormones and help to modulate BPV (Fliser et al. 1998). These effects mediate the changes in BPV that we found from salt as measured by DFA and SampEn.

Our study also showed that beat‐to‐beat blood pressure dynamics, measured by SampEn, and scale exponents *α*_S_ and *α*_L_ were altered with high‐salt diet and the resultant increased systolic and diastolic blood pressure. As *α*_L_ and *α*_S_ measure “roughness” or complexity of the BP pattern over different time scales, we suggest that there are at least two controllers acting over different time scales of BP variation in these rats. *α*_S_ probably represents respiratory oscillations (Peng et al. 1995) and *α*_L_ hormonal influences although further study is needed to evaluate this. Other studies have shown that entropy measures of hormone levels differentiate between health and disease (Hoyos et al. 2014; Norman et al. 2014; Roelfsema and Veldhuis 2016). Our results suggest the potential utility of DFA and SampEn of BP time series to not only detect pathological BP states, but also suggest the time scales over which the controllers act.

Decreased BP complexity indicative of more regular patterns has been previously observed in diseased populations. The study by Subramaniam et al. on patients with preoperative major adverse events during surgery and the study by Vandendriessche et al. on mice after induced shock, measured complexity by multiscale entropy. Both studies showed a loss of complexity as measured by lower multiscale entropy in the diseased groups (Subramaniam et al. 2014; Vandendriessche et al. 2014). In another study, Cerutti et al. studied 20 male patients admitted to the hospital for programmed electrical cardioversion for persistent atrial fibrillation, at rest, and during tilt table testing. They compared the SampEn and ApEn of the systolic and diastolic blood pressure series and showed that both complexity measures increased during tilt in patients in whom the SBP appropriately increased (Cerutti et al. 2014). Shin et al. compared the ApEn of the daytime blood pressures in patients with complicated and uncomplicated hypertension to normotensive patients and showed that ApEn was lower in hypertensive patients compared to controls (Shin et al. 2010). A study by Soehle et al. assessed the complexity of the intracranial pressure in patients with traumatic brain injury during periods of intracranial hypertension and showed loss of complexity in that the SampEn and multiscale entropy of the intracranial pressure time series decreased with intracranial hypertension (Soehle et al. 2013). Nevertheless, not all studies show decreased complexity with pathology, for example, Zhang et al. found increased cross SampEn in a rat model of hemorrhage (Zhang et al. 2007).

The main limitation of our study is that only 15 SS Dahl rats were studied. Other rat strains, breeds, or models of salt‐sensitive hypertension may have different physiologic controllers of blood pressure and produce different DFA and SampEn values. Also, with only 15 animals our chance of finding a spurious finding is increased. Further study is needed with a greater variety of breeds and more animals. Finally, DFA can be biased at short data lengths, particularly 64 or 128 points (Delignieres et al. 2006).While our study used 626–953 points, decreasing the risk of bias, it does not eliminate it and additional studies should be done using longer time series.

One of the strengths of our study is that we measured BPV by two complementary, but not redundant, techniques. DFA has the advantage of being able to estimate the “roughness” of the pattern over different timescales, while SampEn assesses the predictability of the next member of the series across the whole time series. As neural, hormonal, and other controllers of BP have different oscillatory or pulsatile frequencies, DFA may provide a useful tool for determining the interactions between salt or other inputs and the neural, hormonal, and other controllers of BP by matching the DFA scale exponents to the frequency of the controller. Previous studies also showed increased scale exponents coefficients in unhealthy individuals. However, while most of these studies were focused on heart rate time series and not blood pressure time series BP and heart rate are intricately, although not perfectly, linked. Changes in heart rate rhythm lead to beat‐to‐beat changes in stroke volume, which is proportional to BP through vascular compliance. However, over different time scales other factors may contribute to a loss of coupling (Porta et al. 2015). A study by Zhang et al. examined BPV using DFA scale exponents in preterm infants with and without intraventricular hemorrhage. The study included 30 infants and showed a significant increase in the short‐term correlation coefficient *α*_S_ in infants who developed intraventricular hemorrhage while *α*_L_ was unchanged (Zhang et al. 2013). Two studies by Lee et al. on EEG time series data also showed lower scaling exponents in patients with sleep apnea compared to healthy subjects and in unmedicated unipolar depressed subjects compared to nondepressed controls (Lee et al. 2004, 2007). While DFA exponents clearly discriminate between healthy and unhealthy subjects, they do so in varied fashions and to different extents, which indicates that more research on DFA and BPV is needed.

The significance of the current studies is the quantitative demonstration of the role of salt‐induced hypertension, a result of intravascular volume expansion, in modifying the characteristics of BPV, as quantitated by two measures of beat‐to‐beat complexity – SampEn and DFA scaling exponents. Averaged over years or decades, individuals on high‐salt diets would be expected to spend a significantly greater proportion of time under conditions of increased vascular stress and higher intracapillary pressures and wall tension. Clinical monitoring of BPV by nontraditional indices such as those described here may provide earlier indication of subtle changes in intravascular filling and pressure many years before the onset of hypertension and its resultant vascular injury and end‐organ damage. This hypothesis, however, requires prospective validation including the design of long‐term studies on large populations of susceptible individuals.

## Conclusion

Using an animal model, our study is the first study to look mutually at entropy and DFA parameters in hypertensive and nonhypertensive subjects switching from a low‐ to a high‐salt diet. In summary, we showed that hypertensive rats are more susceptible to high‐salt diet with both a greater rise in blood pressure and more reduced beat‐to‐beat BP complexity. Entropy and DFA scaling exponents detected inherent differences in BP variability with hypertension and salt diet that are not observed with the simpler traditional variability measures (SD, CV). Both salt‐sensitive (SS) and ‐protected (SSBN13) rats showed altered BP dynamics with high‐salt versus low‐salt diet, albeit in varied fashion and to different extents. Comparable mean pressures in sensitive and protective rats when on low‐salt diet coupled suggest a protective role of low‐salt intake in hypertensive rats. Moreover, the finding that a low‐salt diet potentially equalizes complexity (entropy and DFA scaling exponents) of beat‐to‐beat BPV in hypertensive and nonhypertensive rats provides evidence that its salutary effects likely act through better coupling of biologic oscillators.

## Conflict of Interest

None declared.

## Supporting information




**Figure S1.** DFA short‐ and long‐term correlations of the SBP of the nine salt‐sensitive rats.
**Figure S2.** DFA short‐ and long‐term correlations of the DBP of the nine salt‐sensitive rats.
**Figure S3.** DFA short‐ and long‐term correlations of the SBP of the six salt‐protected rats.
**Figure S4.** DFA short‐ and long‐term correlations of the DBP of the six salt‐protected rats.
