# Supplementary material for: Effect of salt intake on beat‐to‐beat blood pressure nonlinear dynamics and entropy in salt‐sensitive versus salt‐protected rats
Source: Physiol Rep. 2016 Jun 10;4(11):e12823. doi: 10.14814/phy2.12823 (PMC4908498; doi:10.14814/phy2.12823)

Online Supplemental Material

Effect of Salt Intake on Beat-to-Beat Blood Pressure Nonlinear Dynamics and Entropy in Salt Sensitive versus Protected Rats

Souha A. Fares, PhD[1], Joseph R. Habib, BS[2], Milo C. Engoren, MD[3], Kamal F. Badr, MD[2], and Robert H. Habib, PhD[2,4]

[1]Hariri School of Nursing, American University of Beirut, Beirut - Lebanon
[2]Vascular Medicine Program and Department of Internal Medicine, American University of Beirut, Beirut - Lebanon
[3]Department of Anesthesiology, University of Michigan, Ann Arbor, MI, USA.
[4]Outcomes Research Unit – Clinical Research Institute, American University of Beirut, Beirut - Lebanon

<u>Corresponding Author</u>

Robert H. Habib, PhD

American University of Beirut-Medical Center

PO BOX: 11-0236, Riad El Solh, 1107 2020, Beirut, Lebanon

E-mail: rh106@aub.edu.lb

All authors have no conflict of interest to disclose

Research funded by departmental and Institutional funds.

Presented in part as a Poster at the 2015 Experimental Biology Meeting, Boston, MA, March, 2015.

Figure S1: DFA Short- and long-term correlations of the SBP of the 9 salt-sensitive rats

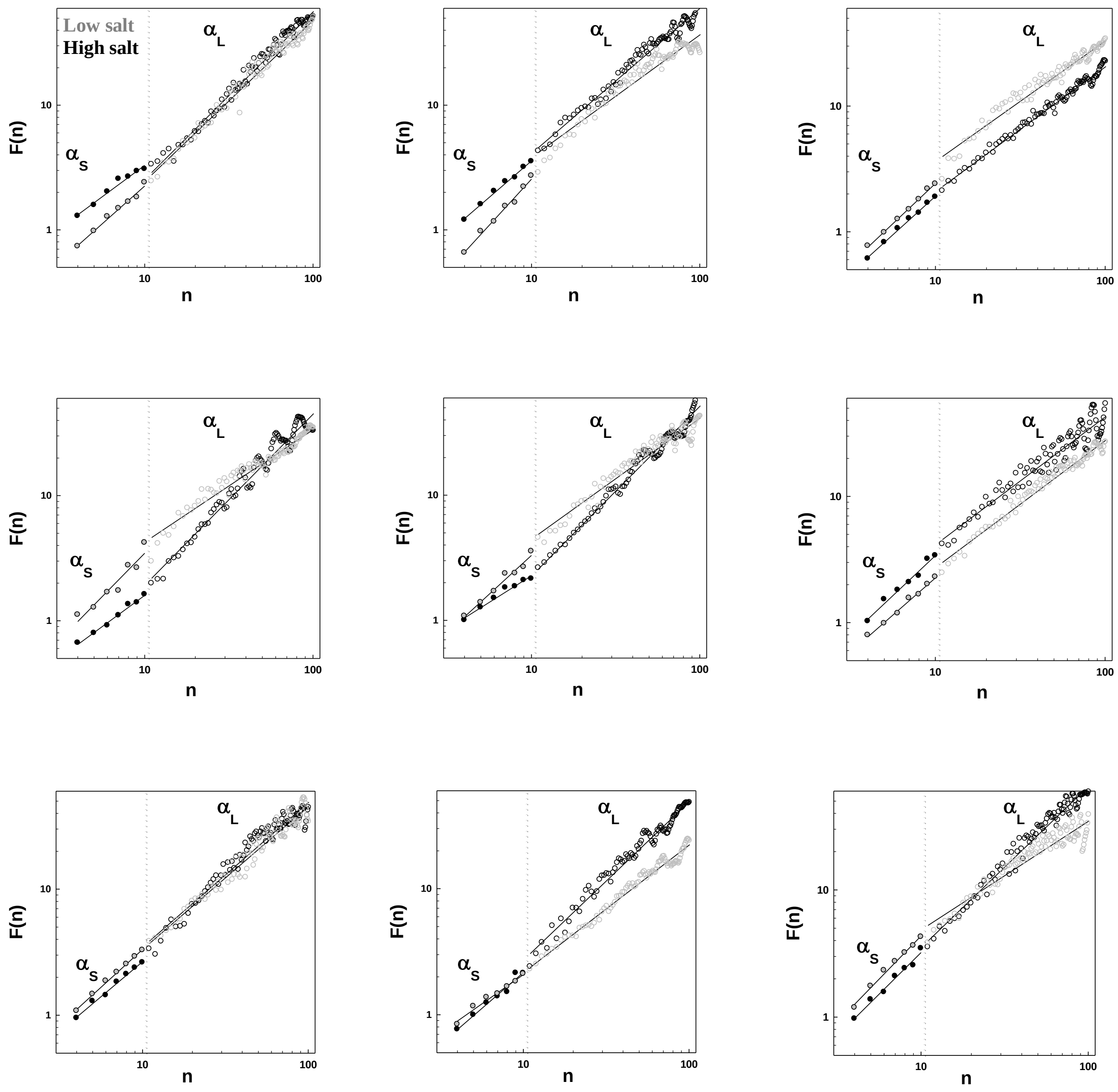

Figure S2: DFA Short- and long-term correlations of the DBP of the 9 salt-sensitive rats

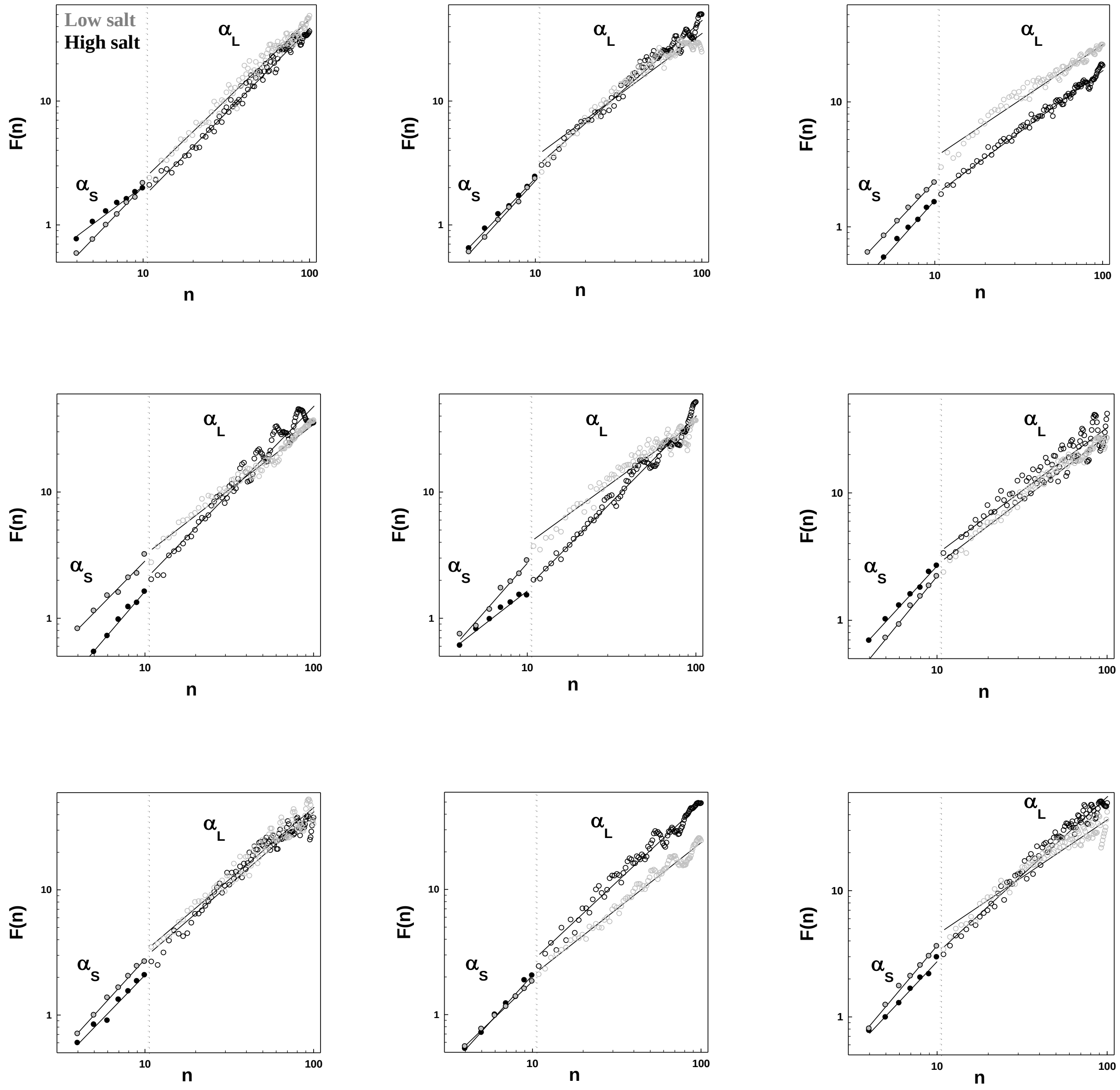

Figure S3: DFA Short- and long-term correlations of the SBP of the 6 salt-protected rats

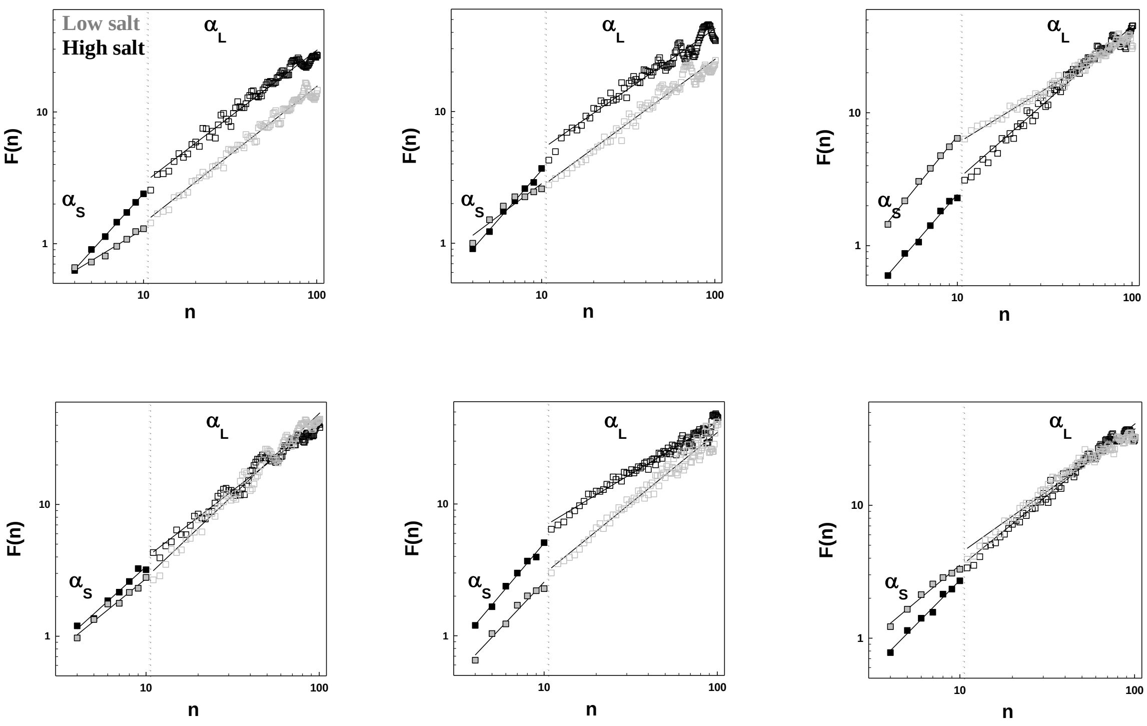

Figure S4: DFA Short- and long-term correlations of the DBP of the 6 salt-protected rats

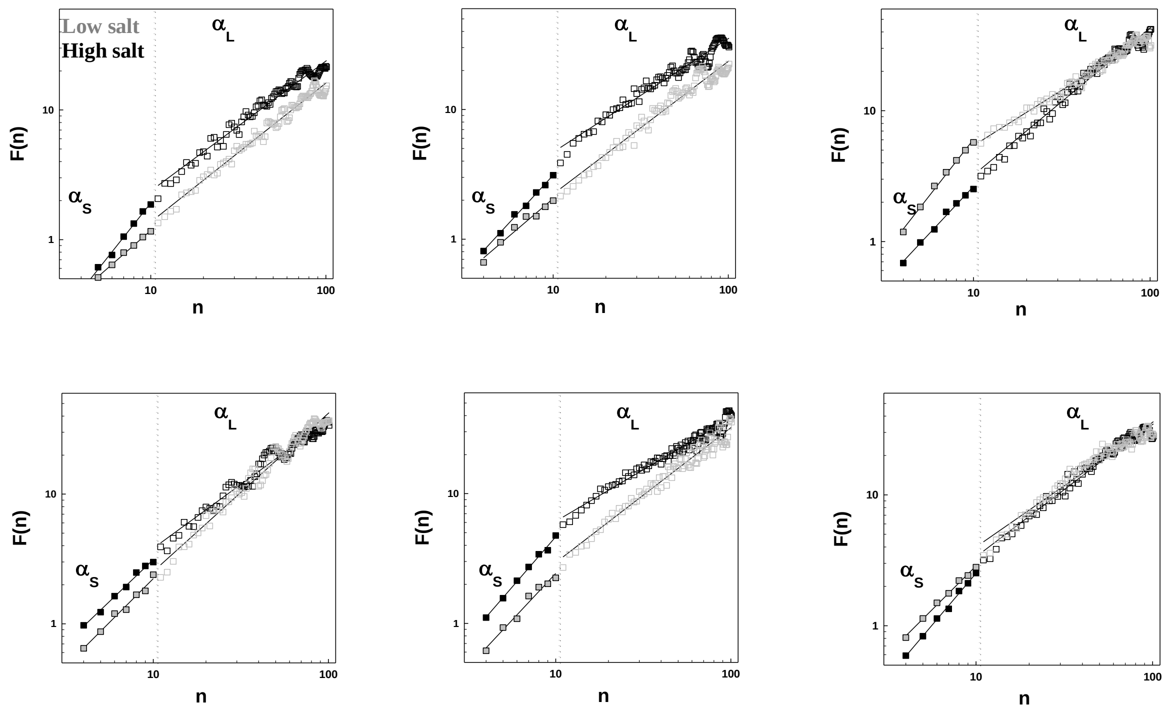

Supplement: Supplementary file 1 — Figure S1. DFA short‐ and long‐term correlations of the SBP of the nine salt‐sensitive rats. Figure S2. DFA short‐ and long‐term correlations of the DBP of the nine salt‐sensitive rats. Figure S3. DFA short‐ and long‐term correlations of the SBP of the six salt‐protected rats. Figure S4. DFA short‐ and long‐term correlations of the DBP of the six salt‐protected rats. [file PHY2-4-e12823-s001.pdf]
